# Proteome and Peptidome Changes and Zn Concentration in Chicken after In Ovo Stimulation with a Multi-Strain Probiotic and Zn-Gly Chelate: Preliminary Research

**DOI:** 10.3390/cimb46020080

**Published:** 2024-02-01

**Authors:** Artur Ciszewski, Łukasz S. Jarosz, Katarzyna Michalak, Agnieszka Marek, Zbigniew Grądzki, Jacek Wawrzykowski, Bartłomiej Szymczak, Anna Rysiak

**Affiliations:** 1 Department of Epizootiology and Clinic of Infectious Diseases, Faculty of Veterinary Medicine, University of Life Sciences in Lublin, Głęboka 30, 20-612 Lublin, Poland; artur.ciszewski@up.lublin.pl (A.C.); zespollukasza@gmail.com (K.M.);; 2 Sub-Department of Preventive Veterinary and Avian Diseases, Faculty of Veterinary Medicine, Institute of Biological Bases of Animal Diseases, University of Life Sciences in Lublin, Głęboka 30, 20-612 Lublin, Poland; agnieszka.marek@up.lublin.pl; 3 Department of Biochemistry, Faculty of Veterinary Medicine, University of Life Sciences in Lublin, Głęboka 30, 20-612 Lublin, Poland; 4 Sub-Department of Pathophysiology, Department of Preclinical of Veterinary Sciences, Faculty of Veterinary Medicine, University of Life Sciences in Lublin, Głęboka 30, 20-612 Lublin, Poland; bartlomiej.szymczak@up.lublin.pl; 5 Department of Botany, Mycology, and Ecology, Maria Curie-Skłodowska University, Akademicka 19, 20-033 Lublin, Poland; anrysiak@o2.pl

**Keywords:** chicken, in ovo, multi-strain probiotic, zinc glycine chelate, MALDI—TOF MS

## Abstract

The aim of the study was to determine differences in the proteome and peptidome and zinc concentrations in the serum and tissues of chickens supplemented with a multi-strain probiotic and/or zinc glycine chelate in ovo. A total of 1400 fertilized broiler eggs (Ross × Ross 708) were divided into four groups: a control and experimental groups injected with a multi-strain probiotic, with zinc glycine chelate, and with the multi-strain probiotic and zinc glycine chelate. The proteome and peptidome were analyzed using SDS-PAGE and MALDI—TOF MS, and the zinc concentration was determined by flame atomic absorption spectrometry. We showed that in ovo supplementation with zinc glycine chelate increased the Zn concentration in the serum and yolk sac at 12 h post-hatch. The results of SDS-PAGE and western blot confirmed the presence of Cu/Zn SOD in the liver and in the small and large intestines at 12 h and at 7 days after hatching in all groups. Analysis of the MALDI—TOF MS spectra of chicken tissues showed in all experimental groups the expression of proteins and peptides that regulate immune response, metabolic processes, growth, development, and reproduction.

## 1. Introduction

Intensive breeding of highly productive chickens brings many problems that can threaten the health and welfare of birds. As a result, animal husbandry becomes less efficient. Promoting sustainable poultry production can minimize these negative effects [1,2,3,4]. A proven way to improve the performance of broiler chickens, product quality, and health is to optimize nutrition [5,6]. Studies have shown the importance not only of appropriate feeding of birds from hatching to slaughter, but especially of in ovo feeding [7].

Among various bioactive substances administered in ovo, increasing importance is currently ascribed to multi-strain probiotic and organic forms of mineral compounds, specifically chelates [8,9,10]. Among multi-strain probiotics, formulations containing effective microorganisms (EM) are particularly worthy of attention [11]. Supplementation of poultry feed with EMs has been shown to stimulate immunity in chickens [11] and contribute to the synthesis of various biologically active compounds, including enzymes and vitamins [12]. These processes increase daily weight gains, improve feed digestibility, increase productivity, and reduce mortality [13,14]. The use of EM probiotics as feed additives also increases intestinal assimilation of elements by creating an acidic environment in the gut [15]. Combined supplementation with a multi-strain probiotic and an easily assimilated element in chelated form guarantees a more efficient impact on the body [11]. In addition, supplementation with probiotics, including multi-strain probiotics, during embryonic development has been shown not only to modify the composition of the microbiome of the developing and newly hatched chick, but also to influence metabolism during post-hatch development [16].

In addition to probiotics, organic chelated forms of elements are increasingly used in the diet of broiler chickens. These include Zn-Gly chelate, administered mainly due to the high bioavailability of Zn in combination with glycine [17,18]. Zn-Gly has been shown to have direct or indirect impact on birds, e.g., by modifying the immune response [19,20] or stimulating growth and development [21]. Literature data also confirm the beneficial effect of zinc on metabolic processes in animal tissues [22]. Moreover, zinc also influences the fatty acid profile and regulates transport of intestinal lipids and the metabolism of prostaglandins [23,24]. Additional in ovo administration of zinc increases its concentration and resorption by the embryo, which positively affects health and production parameters in the post-hatch period [25]. Zn absorbed by the embryo accumulates mainly in the liver and takes part in building the tissues of the developing chick [26,27].

It is also worth noting that the gastrointestinal tract (GIT) microbiota and processes regulating the expression of genes responsible for metabolism undergo modulation in early embryonic development [28]. In ovo supplementation with a multi-strain probiotic and Zn chelate during this period may contribute to embryonic programming, affecting the entire body during the post-hatch period. Moreover, modulation of the bacterial microbiota of chicks in the embryonic stage influences amino acid transporters and ion transporters, which are associated with various bacterial strains making up the intestinal microbiome of broilers [29]. Microorganisms that constitute the microbiome and probiotic supplemented in ovo and in vivo can influence the metabolism of minerals such as calcium, iron, magnesium, selenium, potassium, copper, zinc, and others by increasing or inhibiting their absorption through the digestive tract [30]. This happens through interactions between microorganisms, minerals, and host cells [31]. In particular, probiotics increase the bioaccessibility and bioavailability of Zn by influencing the expression of specific proteins involved in the transport of Zn through the intestinal wall [30]. Examples of such interactions include increasing the level of metallothionein in enterocytes through the production of short-chain fatty acid (SCFA), which lowers intestinal pH and increases the solubility of Zn, or the biotransformation of inorganic Zn to its organic form by specific probiotic strains [32]. Therefore, supplementation with probiotics may result in increased Zn absorption and its accumulation in the body.

Changes in the microbiome through in ovo supplementation may also lead to metabolic changes, which may also influence the growth of chicks after hatching. The beneficial effect of probiotics and zinc on health and production parameters in chickens is undoubtedly related to changes in the number and activity of specific proteins involved in immune and metabolic processes. Zn^2+^ is known to affect the proteolytic activity of transthyretin (TTR) and may cause structural changes in this protein and thus in its stability and aggregation [33]. Therefore, high Zn intake in feed may, on the one hand, stimulate overall hepatocyte metabolism when TTR is synthesized, while, on the other hand, it may cause pathological changes leading to the development of diseases involving TTR [33]. An increased supply of zinc in the body also leads to an increase in the concentration of metallothionein (MT) in the liver and the activation of many enzymes, e.g., glutamate dehydrogenase (GDH), which is involved in the Krebs cycle [34]. Data in the available literature indicate that the use of various forms of zinc as a supplement causes an increase in the expression of genes in the liver that participate in the response to oxidative stress, as well as an increase in the activity of antioxidant enzymes and a reduction in lipid peroxidation processes [35,36]. These data clearly confirm the beneficial effect of zinc on metabolic processes in hepatocytes. Simultaneously, probiotic microbes can facilitate absorption of microelements such as Zn [30], so understanding these relationships in quantitative and qualitative terms will create the basis for a rational approach to effective poultry feeding using dietary supplements and in ovo technology. The aim of the study was to determine differences in the proteome and peptidome and zinc concentrations in the serum and tissues of chickens which have received a multi-strain probiotic and/or Zn-Gly chelate in ovo. Presumably, these analytical methods will not only enable visualization of the proteome and peptidome of chicken tissues, but may also reveal any differences in protein expression depending on the time of analysis and the use of in ovo supplements. The results can serve a basis for further research involving the identification of specific proteins differentiating various groups and their post-translational modifications, which will make it possible to learn which proteins appear in the body or undergo increased synthesis as a result of the use of these supplements.

## 2. Materials and Methods

### 2.1. Ethical Statement

The experiment was conducted at the Experimental Station of the Poznań University of Life Sciences, Gorzyń 4, Międzychód commune. Consent for all research procedures was obtained from the Local Ethics Committee for Animal Testing at the University of Life Sciences in Lublin, Poland (approval no. 106/2022 of 17 October 2022).

### 2.2. Incubation Period

Hatching eggs were collected from Ross × Ross 708 broilers at the age of 36 weeks (15th week of laying) and transported to the Experimental Station of the Poznań University of Life Sciences. Eggs were stored at 21 °C for 24 h before being incubated. After they were weighed and labelled, the eggs were disinfected with Viron FF (glutaraldehyde, didecyldimethylammonium chloride, quaternary ammonium compounds, and benzyl-C12-C16-alkyldimethyl; DDD-1, Bielsko Biała, Poland) in 1 mL of L-1 solution and then placed in JARSON Model JD-18 incubators (Gostyń, Poland) at 37.8 °C and a relative humidity of 55–60% (days 1–18). On the 19th day of incubation (DOI), the eggs were transferred to a hatching chamber (JARSON Model ATLAS-180, Gostyń, Poland). Relative humidity was maintained at 60–65% for the last three days of incubation (19–21 DOI). Eggs were candled at 7 and 17 DOI, and unfertilized and dead embryonic eggs were discarded.

### 2.3. In Ovo Inoculations

At 17 DOI, 1400 fertilized eggs of similar weight were divided randomly into four groups, with 10 replicates per group and 35 eggs per replicate (350 eggs per group). The four groups were as follows: eggs injected with sterile 0.9% physiological saline solution— control group I (Zn-Gly-Prob-); eggs injected with a multi-strain probiotic (EM Provet; Greenland EM Technology, Janowiec, Poland)—group II (Prob+); eggs injected with zinc glycine chelate (ARKOP Sp. z o.o., Bukowno, Poland)—group III (Zn-Gly+); and eggs injected with the multi-strain probiotic and zinc glycine chelate—group IV (Zn-Gly+Prob+). The multi-strain probiotic was in the form of a powder. One gram of multi-strain probiotic contains the following strains: *Saccharomyces cerevisiae* (Y200007) 5 × 10^6^ CFU, *Lactobacillus casei* (ATCC 7469) 5 × 10^8^ CFU, and *Lactobacillus plantarum* (ATCC 8014) 5 × 10^8^ CFU. The multi-strain probiotic was dissolved in PBS, and 100 µL of this dilution contains 1 × 10^5^ CFU *S. cerevisiae*, 1 × 10^7^ CFU *L. casei*, and 1 × 10^7^ CFU *L. plantarum.* Zn-Gly powder (250 mg Zn-Gly per g of product) was dissolved in deionized water. The resulting solutions contained 100 µg Zn-Gly/100 µL MQ water. All procedures are described in Ciszewski et al. [11].

Prior to inoculation, at 17 DOI, all eggs were decontaminated with 75% ethanol, and within 30 min, all eggs were inoculated through the air chamber (using a 2.5 cm-long 23G needle) with 500 μL of 0.9% saline or a bioactive compound to the amniotic sac. The puncture sites in the eggs were sealed with paraffin. The eggs were then placed in an incubator. All in ovo methods are described by Alizadeh et al. [37].

### 2.4. Birds and Housing

One day after hatching, 150 chicks were selected from each group (10 replicates of 15 birds per replicate from each group). The experimental period was 7 days. The birds were fed a basic starter S mixture (1–7 days) in the form of crumble according to nutritional recommendations for broiler chickens by Ross 308 (Aviagen, Broiler Nutrition Specifications, https://aviagen.com/eu/brands/ross/products/ross-308, accessed on 12 September 2022). No bioactive compounds, coccidiostats, or antibiotics were added to the diet. The content of nutrients in the feed was calculated from the chemical composition of the feed ingredients and the metabolic energy value. Composition and nutrient value of basal diet (%) are described in detail in work by Ciszewski et al. [11]. The chickens were kept under controlled conditions as recommended for this line. They were reared on wood chips in aviaries with controlled temperature and humidity, with access to feeding lines and nipple drinkers. The light intensity was 30 lux with a 22L:2D photoperiod. The temperature was kept at 31–33 °C and the relative humidity was kept at 60% ± 10%.

### 2.5. Blood and Tissue Sample Collection

Blood and tissues were sampled from three birds per replicate (30 samples in total) in each group. The samples were not pooled. Blood samples were collected at 12 h post-hatch from chicks sacrificed by decapitation (before feeding) and again at 7 days post-hatch. Blood was collected into tubes with a clotting activator (Medlab Products, Raszyn, Poland) and transported to the laboratory within 1 h. Then, the samples were centrifuged at room temperature (20–22 °C) for 15 min at 1000× *g*, and the serum was stored at –80 °C. 

At the same time as the blood collection, i.e., at 12 h and 7 days post-hatch, tissues from the liver, small intestine (ileum), and large intestine (between the cloaca and the entrance to the caecum) were collected for analysis of zinc concentrations and proteomic analysis. The yolk sac was collected only at 12 h after hatching for analysis of zinc concentrations and proteomic analysis. Decapitation was performed according to the procedures described in the AVMA Guidelines for the Euthanasia of Animals (https://www.avma.org/resources-tools/avma-policies/avma-guidelines-euthanasia-animals, accessed on 12 September 2022) [38]. The tissue samples were collected by dissection immediately after decapitation of the birds. After collecting the tissues were washed in ice-cold saline (87 mM NaCl, 2.5 mM KCl, 1.25 mM NaH_2_PO_4_, 25 mM NaHCO_3_, 0.5 mM CaCl_2_, 7 mM MgSO_4_, 25 mM glucose, 75 mM sucrose, pH 7.4) and stored at −80 °C for further analysis.

### 2.6. Determination of the Zinc Concentration in Serum and Tissues by Flame Atomic Absorption Spectrometry (FAAS)

The Zn concentration was determined 12 h after hatching and 7 days after hatching in samples of the liver, small intestine (ileum), and serum of the birds in the control (I) and experimental (II–IV) groups. The Zn concentration was also determined at 17 days of incubation and 12 h after hatching in the yolk sac in all groups. Samples of 0.5 g were weighed out to within 1 mg and subjected to wet mineralization with concentrated nitric acid (HNO3) in a closed-loop microwave system using a CEM Mars Xpress digestion oven (Matthews, NC, USA). The following parameters were used in the mineralization process: magnetron power (W)—800, temperature rise time (minutes)—25, temperature (°C)—210, and time of temperature maintenance (minutes)—15. The zinc concentration was determined by atomic absorption spectrometry using a Varian SpektrAA 280FS spectrometer with an SPS-3 autosampler and SIPS diluter (Belrose, Australia), with an air-acetylene flame. Validation parameters are presented in Table 1.

### 2.7. MALDI—TOF MS

Tissue homogenates were obtained by short homogenization on ice with the addition of a protease inhibitor cocktail (Mass Spectrometry Safe Protease and Phosphatase Inhibitor Cocktail; Sigma-Aldrich, Poznań, Poland). Homogenates were centrifuged (5000× *g*, 20 min, 4 °C), and supernatants were collected. Tissue homogenates were purified and concentrated by ZipTip with 0.6 µL C18 resin using 100% acetonitrile (ACN) as the wetting solution and 0.1 TFA/50% ACN for elution. Eluted samples were spotted on an Anchor Chip MALDI plate (Bruker, Bremen, Germany) and covered with 1 μL of α-cyano-4-hydroxycinnamic acid matrix (HCCA, Bruker, Bremen, Germany). Simultaneously, a standard solution (Peptide Calibration Standard II, Bruker, Bremen, Germany) was applied to the calibration spots. Mass spectra were recorded using an Ultraflextreme MALDI TOF/TOF (Bruker, Bremen, Germany) spectrometer with linear mode for molecular weights greater than 5000 Da (parameters as follows: linear detector gain: 26 × 2998 V, frequency: 1000 Hz, 2 Gs/s, and mass range: 4000 to 20,000 *m*/*z*) and reflector mode for lower molecular weights (parameters as follows: reflector detector gain: 8.8 × 2531 V, frequency: 1000 Hz, 4 GS/s, and mass range: 700 to 4000 *m*/*z*). All spectra were smoothed and baseline corrected in flexAnalysis 3.0 software (Bruker, Bremen, Germany). The peptide and protein masses shown on the spectra were compared with each other and with UniProt biodata resource (https://www.uniprot.org, accessed on 8 November 2023).

### 2.8. SDS-PAGE Analysis

SDS-PAGE electrophoresis was conducted by a standard procedure by Laemmli [39]. The procedure was carried out in a 10% resolving gel (Tris–HCl buffer with pH 8.8), and 4% polyacrylamide in Tris–HCl buffer with pH 6.8 was used as a stacking gel and Tris–glycine running chamber buffer. Separation took place at a constant current of 100 mA. Additionally, a molecular weight standard (Perfect TM Color Protein Ladder, EurX, Gdańsk, Poland) with a molecular weight range from 7 to 240 kDa was used during separation. Gels were stained with Coomassie blue (Sigma-Aldrich, Poznań, Poland) and silver. The stained gels were scanned by ImageScanner III LabScan 6.0 (GE Healthcare, Chicago, IL, USA).

### 2.9. Statistical Analysis

The data were analyzed with the Shapiro–Wilk test using Statistica 13.2 PL software (StatSoft, Kraków, Poland) to check the normality of the distribution, which was not normal in several cases. A Box–Cox transformation of the data was therefore performed to obtain a normalized distribution and a homogeneous variance. The results were expressed as the arithmetic mean and standard deviation (SD). Parametric tests were used to demonstrate the significance of statistical differences (*p* < 0.05) between the control group (group I) and the experimental groups (groups II–IV) using two-way ANOVA and post hoc Tukey tests. The t test was used to test differences within the same group over time, i.e., at 12 h and at 7 days after hatching. A two-factor analysis of variance modelling approach was used to study the effect of two factors on the average Zn levels in serum and selected tissues of chickens. Four study groups were established: group I—control (Prob-, Zn-Gly-); group II (Prob+); group III (Zn-Gly+); and group IV (Zn-Gly+Prob+), in which the interaction effects between the two factors were statistically analyzed. The measure of the effect was the coefficient η^2^ (eta), which determines the proportion of the total variance explained by the analyzed factors (fix effect of the model). This was calculated using the following formula:

η^2^ = SS_efekt_/SS_total_ × 100%,

where SS_efekt_—sum of squares of standard deviations associated with the effect and SS_total_—total sum of squares for the effects + SS_error_.

The results were presented in graphic form, with the same letter designations indicating the absence of statistically significant differences. Capital letters were used to show statistically significant results between groups in time (*t* test), and lower-case letters to indicate differences shown in ANOVA and post hoc tests.

The volumes of SOD bands were expressed as arithmetic mean and standard deviation. Analysis of variance (ANOVA) and Tukey’s HSD test were performed to demonstrate the significance of statistical differences (*p* ≤ 0.05). Student’s *t* test (*p* ≤ 0.05) was used to analyze the differences in the average volume of the band in a given organ between 12 h and 7 days (e.g., liver 12 h group I versus liver 7 days group I).

## 3. Results

### 3.1. Zn Concentration in the Serum, Liver, Small Intestine, and Yolk Sac

Two-way analysis of variance (ANOVA) was performed to determine the effect of and interaction between the two in ovo supplements, zinc-glycine chelate (Zn-Gly) and the multi-strain probiotic, and their effect on zinc levels in the tissues (Table 2).

**Figure 1:**
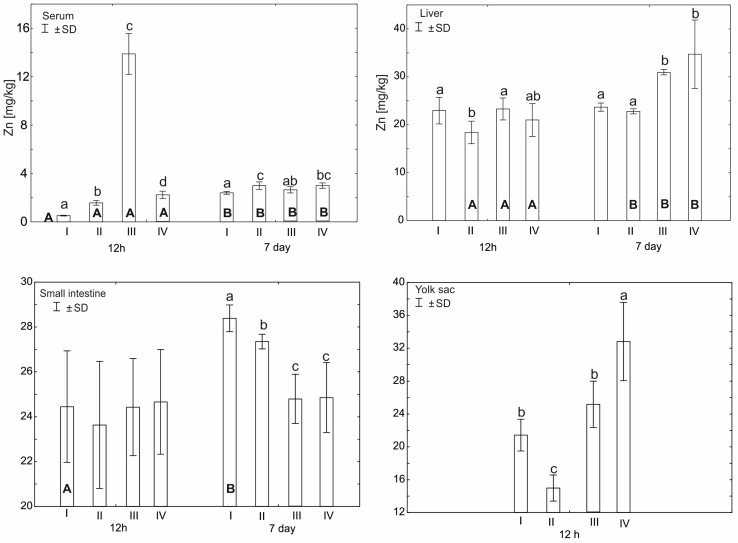
Comparison of zinc concentration (mean ± SD) for the tissues examined during two periods: 12 h and 7 days after hatching (capital letters—A, B) according to the *t* test (*p* ≤ 0.05) and between four experimental groups at the same time: group I—control group (Zn-Gly-Prob-; eggs injected with 0.9% physiological saline solution); group II (Prob+)—eggs injected with a multi-strain probiotic; group III (Zn-Gly+)—eggs injected with zinc glycine chelate; group IV (Zn-Gly+Prob+)—eggs injected with a multi-strain probiotic and zinc glycine chelate (lower-case letters—a, b, c, d). Different letters indicate statistically significant differences using two-way analysis of variance (ANOVA).

Statistically significant differences in plasma zinc concentration 12 h after hatching (*p* ≤ 0.05) were observed between group III and other experimental groups. Statistically significant differences (*p* ≤ 0.05) in zinc concentration were also observed between groups I (control) and II and IV. After 7 days from hatching, a statistically significant difference (*p* ≤ 0.05) in zinc concentration was observed in group I compared to groups II and IV. Statistically significant differences (*p* ≤ 0.05) in serum zinc levels were also observed in all groups between 12 h and 7 days after hatching (Figure 1).

Statistically significant differences in zinc content in the liver 12 h after hatching (*p* ≤ 0.05) were observed in group II compared to group III and group I. However, after 7 days from hatching, statistically significant differences (*p* ≤ 0.05) in the level of the tested microelement were observed in groups IV and III in relation to groups II and I. Statistically significant differences (*p* ≤ 0.05) in the level of zinc were also observed on the seventh day after hatching in groups I–IV, compared to the level of zinc in these groups 12 h after hatching (Figure 1).

Statistically significant differences (*p* ≤ 0.05) in zinc content in the small intestine, ileum, were observed 7 days after hatching in group I compared to groups II, III, and IV. Statistically significant differences (*p* ≤ 0.05) in the level of zinc in the small intestine between the 12th hour after hatching and the seventh day were observed only in group I (Figure 1).

On day 17 of incubation, the average level of Zn in yolk sac, before in ovo supplementation, was 27.8 ± 0.79 mg/kg. Comparing this result with the level of Zn 12 h after hatching, a statistically higher level of zinc was recorded in group IV and was lower in groups I–II. Statistically significant differences (*p* ≤ 0.05) in the level of zinc in the yolk sac 12 h after hatching were observed in group II compared to groups I, III, and IV. Detailed data are presented in Figure 1.

A two-factor analysis of variance (ANOVA) was also performed, in order to determine the effect and interaction between the two dietary supplements, zinc-glycine chelate (group III, Zn-Gly+) and multi-strain probiotic (group II, Prob+), and their effect on zinc levels in the serum and tissues. This was due to the administration of the supplement mixture to individuals in experimental group IV (Zn-Gly+Prob+) (Figure 1, Table 2 and Table S1). For serum 12 h after hatching, the administration of Zn-Gly significantly increased Zn levels. The combination of zinc glycine chelate and multi-strain probiotic (group IV, Zn-Gly+Prob+) significantly decreased zinc levels. After 7 days, a significant effect of increasing Zn levels was observed for the multi-strain probiotic. Both Zn-Gly+ and Zn-Gly+Prob+ did not significantly affect tissue zinc levels in serum. In liver cells, the reduction in Zn levels 12 h after multi-strain probiotic (group II, Prob+) administration was statistically significant. Zn-Gly+ supplementation after 7 days increased Zn levels in liver cells to a similar extent as supplementation with mixtures of Zn-Gly+Prob+. The combination of supplements did not produce statistically significant effects. In small intestine cells, a significant effect of the supplements was not observed until the 7th day after hatching; it was most visible after supplementation with multi-strain probiotic, while the administration of Zn-Gly+ and Zn-Gly+Prob+ significantly reduced zinc levels. A mixture of Zn-Gly+Prob+ supplements significantly improved zinc absorption in the yolk sac, where zinc levels were highest after 12 h compared to other experimental groups. Detailed data are presented in Table 2 and Table S1.

### 3.2. Peptide Profile Analysis

**Figure 2:**
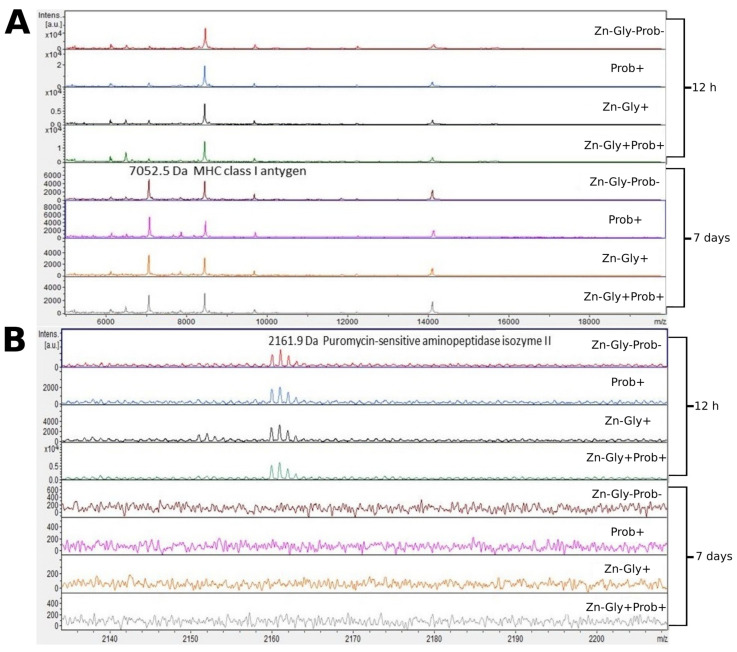
MALDI—TOF MS obtained after enzymatic hydrolysis of the liver of chickens during two periods: 12 h and 7 days after hatching. (**A**) mass ranges from 6000 to 18,000; (**B**) mass ranges from 2140 to 2200. Group I—control group (Zn-Gly-Prob-; eggs injected with 0.9% physiological saline solution); group II (Prob+)—eggs injected with a multi-strain probiotic; group III (Zn-Gly+)—eggs injected with zinc glycine chelate; group IV (Zn-Gly+Prob+)—eggs injected with a multi-strain probiotic and zinc glycine chelate.

**Figure 3:**
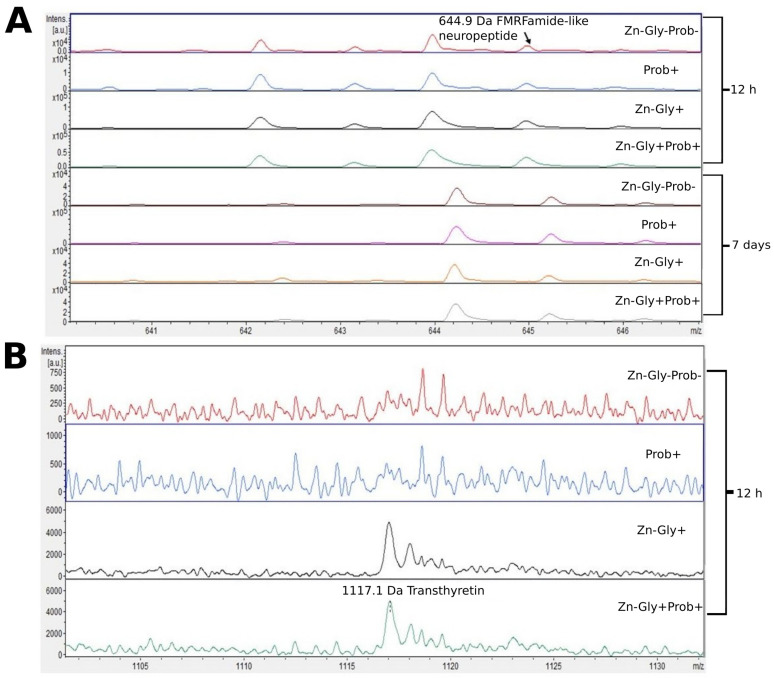
MALDI—TOF MS obtained after enzymatic hydrolysis of the liver of chickens during two periods: 12 h and 7 days after hatching. (**A**) mass ranges from 641 to 645; (**B**) mass ranges from 1105 to 1130. Group I—control group (Zn-Gly-Prob-; eggs injected with 0.9% physiological saline solution); group II (Prob+)—eggs injected with a multi-strain probiotic; group III (Zn-Gly+)—eggs injected with zinc glycine chelate; group IV (Zn-Gly+Prob+)—eggs injected with a multi-strain probiotic and zinc glycine chelate.

We showed differences between groups by comparing the intensities of mass spectra. MALDI—TOF MS identified 12 proteins (Table 3). The peptide profiles of liver homogenates were checked using a mass spectra range of 600–10,000 Da (Figure 2). The following proteins were identified in the liver peptide profile: FMRFamide-like neuropeptide, with a molecular weight of 645 Da, transthyretin (1117 Da), puromycin-sensitive aminopeptidase isozyme II (2162 Da), and MHC class I antigen (7053 Da) (Table 3). Analysis of the differences within groups at 12 h and 7 days post-hatch revealed the absence of several peptide signals. Molecular weights absent in the group at 7 days were 642 and signals were in the range of 2160–2162 *m*/*z* (Figure 2 and Figure 3). Significant differences in the mass spectra of liver homogenate proteins were observed between 12 h and 7 days post-hatch. Ion 8460 *m*/*z* in all samples collected 12 h after hatching was clearly more intense than in samples collected at 7 days after hatching. Only the signal ratio of 7052 to 8460 *m*/*z* differentiated the samples taken at 12 h after hatching and 7 days after hatching (Figure 2). Comparative analysis of peptide profiles in individual groups at 12 h after hatching revealed new peptides in the groups supplemented with multi-strain probiotic+ZnGly and ZnGly (Figure 3).

**Figure 4:**
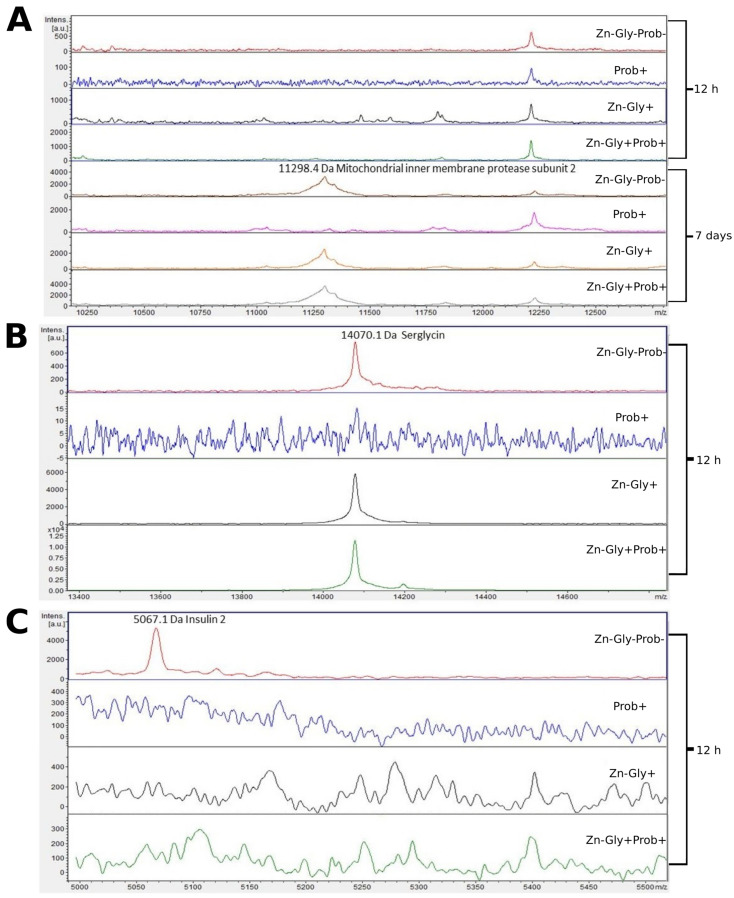
MALDI—TOF MS obtained after enzymatic hydrolysis of the small intestine of chickens during two periods: 12 h and 7 days after hatching. (**A**) mass ranges from 10,250 to 12,500; (**B**) mass ranges from 13,400 to 14,600; (**C**) mass ranges from 5000 to 5500. Group I—control group (Zn-Gly-Prob-; eggs injected with 0.9% physiological saline solution); group II (Prob+)—eggs injected with a multi-strain probiotic; group III (Zn-Gly+)—eggs injected with zinc glycine chelate; group IV (Zn-Gly+Prob+)—eggs injected with a multi-strain probiotic and zinc glycine chelate.

Three proteins were identified in the small intestine peptide profile: insulin 2, with a molecular weight of 5067 Da, mitochondrial inner membrane protease subunit 2 (11,298 Da), and serglycin (14,070 Da) (Figure 4, Table 3). Analysis of the differences between samples taken at 12 h and 7 days post-hatch revealed the formation of new peptides of 11,300 and 13,875 *m*/*z* in all groups except the multi-strain probiotic-supplemented group at 7 days post-hatch (Figure 4). The analysis of molecular weights higher than 5000 *m*/*z* showed that at 12 h post-hatch the 5067 *m*/*z* signal had disappeared only in the control group, while the 14,070 protein peak was absent in group II, supplemented with the multi-strain probiotic (Figure 4).

**Figure 5:**
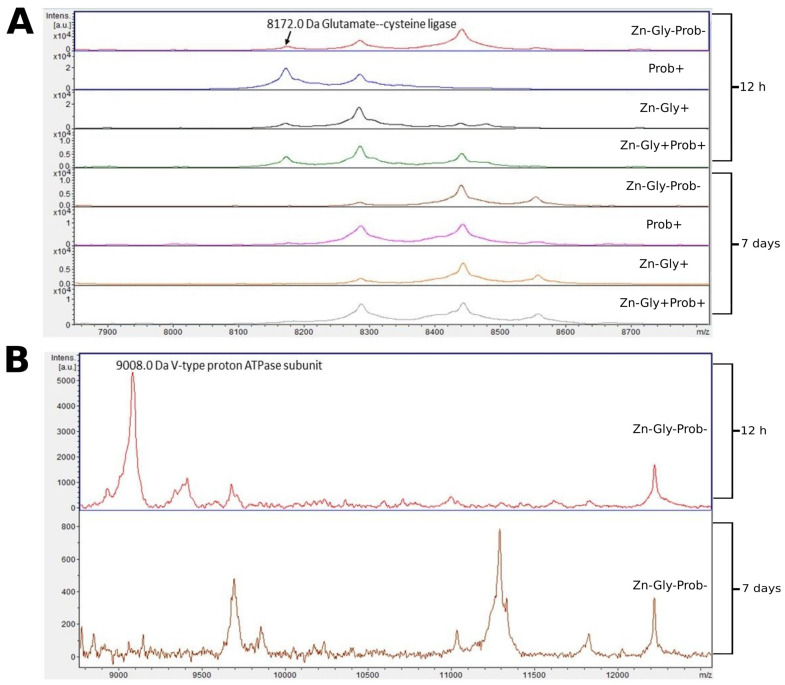
MALDI—TOF MS obtained after enzymatic hydrolysis of the large intestine of chickens during two periods: 12 h and 7 days after hatching. (**A**) mass ranges from 7900 to 8700; (**B**) mass ranges from 9000 to 12,000. Group I—control group (Zn-Gly-Prob-; eggs injected with 0.9% physiological saline solution); group II (Prob+)—eggs injected with a multi-strain probiotic; group III (Zn-Gly+)—eggs injected with zinc glycine chelate; group IV (Zn-Gly+Prob+)—eggs injected with a multi-strain probiotic and zinc glycine chelate.

Two proteins were identified in the peptide profile of the large intestine: glutamate-cysteine ligase, with a molecular weight of 8172 Da, and V-type proton ATPase subunit, with a molecular weight of 9008 Da (Figure 5, Table 3). Comparison of larger mass spectra showed clear differences around 8172 *m*/*z*. This signal was observed at 12 h post-hatch but was absent after 7 days (Figure 5). Differences were also observed in the control group, in which a peak near 9000 *m*/*z* was observed in the large intestine samples at 12 h post-hatch, and a low-intensity mass of 11,250 was observed at 7 days post-hatch (Figure 5).

**Figure 6:**
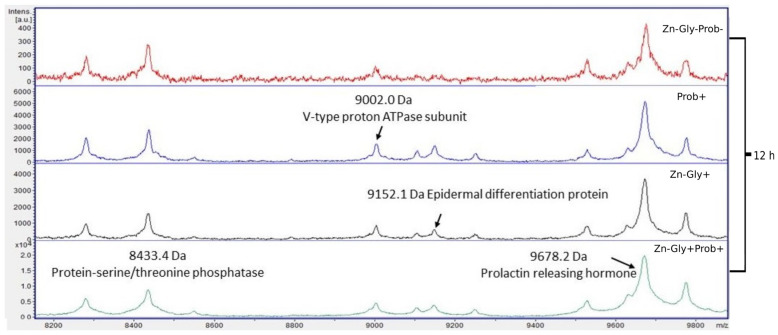
MALDI—TOF MS in the yolk sac of chickens 12 h after hatching. Mass ranges from 8200 to 9800. Group I—control group (Zn-Gly-Prob-; eggs injected with 0.9% physiological saline solution); group II (Prob+)—eggs injected with a multi-strain probiotic; group III (Zn-Gly+)—eggs injected with zinc glycine chelate; group IV (Zn-Gly+Prob+)—eggs injected with a multi-strain probiotic and zinc glycine chelate.

Four proteins were identified within the yolk sac: protein-serine/threonine phosphatase with a molecular weight of 8433 Da, V-type proton ATPase subunit (9002 Da), epidermal differentiation protein (9152 Da), and prolactin-releasing hormone (9678 Da) (Figure 6, Table 3).

### 3.3. Protein Mass Distribution Visualized by SDS-PAGE

**Figure 7:**
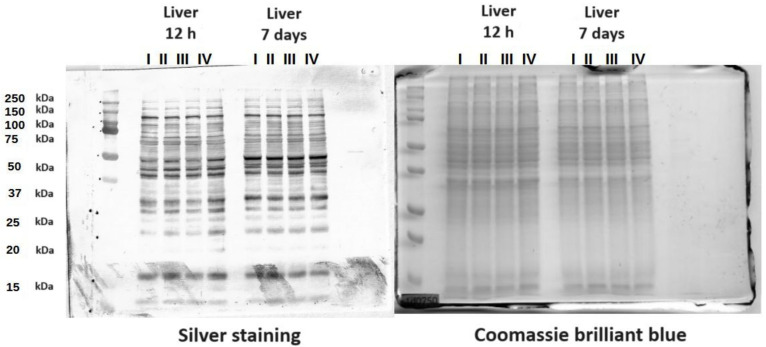
SDS-PAGE protein gels stained with Coomassie brilliant blue and silver. The gels contain proteins from chicken liver. I—control group (Zn-Gly-Prob-; eggs injected with 0.9% physiological saline solution); group II (Prob+)—eggs injected with a multi-strain probiotic; group III (Zn-Gly+)—eggs injected with zinc glycine chelate; group IV (Zn-Gly+Prob+)—eggs injected with a multi-strain probiotic and zinc glycine chelate.

The SDS-PAGE gels presented the molecular weight distribution of proteins in the tissues in the range of 15–250 kDa. The aim of SDS-PAGE electrophoresis is to analyze protein mass fractions in samples. The gels were stained with both silver and Coomassie brilliant blue to expose as many proteins as possible. Proteins in a range from 25 to 150 kDa made up the largest share of the protein composition. There was also a band of around 15 kDa representing the protein Cu/Zn SOD. This band was not present in the yolk sac (Figure 7 and Figures S1–S6).

### 3.4. Analysis of the Presence of Superoxide Dismutase (SOD) in the Liver and in the Small and Large Intestine

**Figure 8:**
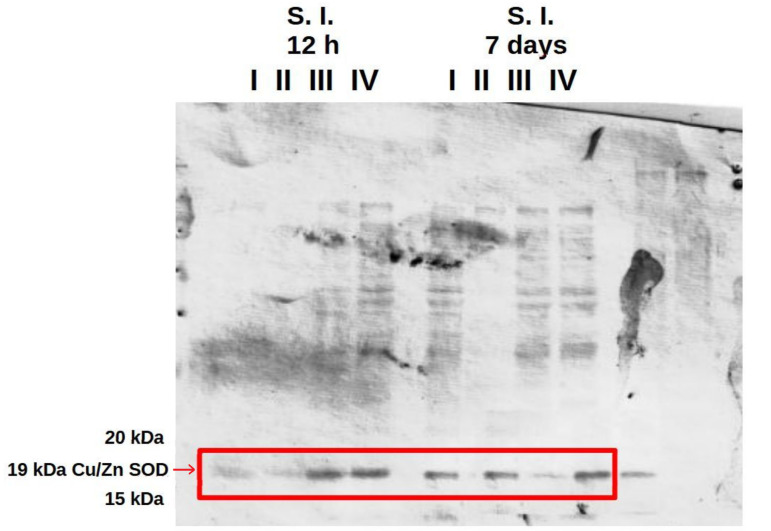
Western blot with SOD band extracted from chicken small intestine. I—control group (Zn-Gly-Prob-; eggs injected with 0.9% physiological saline solution); group II (Prob+)—eggs injected with a multi-strain probiotic; group III (Zn-Gly+)—eggs injected with zinc glycine chelate; group IV (Zn-Gly+Prob+)—eggs injected with a multi-strain probiotic and zinc glycine chelate.

The western blot results confirmed the presence of Cu/Zn SOD in the liver and in the small and large intestine at 12 h and at 7 days after hatching in all experimental groups and the control (Figure 8 and Figures S7–S9). Analysis of polyacrylamide gels using GelAnalyzer 19.1 software showed the highest expression of Cu/Zn SOD in the small and large intestine at 12 h post-hatch in the groups of birds receiving the multi-strain probiotic and Zn-Gly chelate (group IV) or Zn-Gly chelate (group III). At 7 days of age, however, the highest expression of Cu/Zn SOD was noted in the small intestine in group IV, receiving the multi-strain probiotic and Zn-Gly chelate, and in the large intestine in group III, receiving Zn-Gly chelate (Table 4, Figure 8 and Figures S7–S9).

Analysis of the differences in the average volume of the band in a given organ in a given group between 12 h and 7 days (e.g., liver 12 h group I vs. liver 7 days group I), based on Student’s *t* test (significance level *p* ≤ 0.05) showed statistically significant differences between each pair at *p* < 0.00001 (Table 4).

## 4. Discussion

The experiment showed that in ovo supplementation with Zn-Gly chelate increased the Zn concentration in the serum and yolk sac at 12 h post-hatch. The Zn concentration was also increased in the yolk sac in the group of birds receiving a multi-strain probiotic and Zn-Gly chelate in ovo. No changes were noted in the Zn concentration in the intestinal tissues or liver of either group of birds. In contrast, Radi et al. [40] and Ramiah et al. [41] showed increased Zn concentrations in the serum, liver, and muscles of broilers fed a diet with zinc oxide nanoparticles (ZONPs); the increase in the concentration was proportional to the level of zinc supplementation.

In the present study, the group of birds receiving the multi-strain probiotic and Zn-Gly chelate (groups III and IV) showed increased Zn concentrations in the liver at 7 days of age (Table 2, Figure 1). These results demonstrate that the zinc concentration in the birds remains adequate for normal development and metabolism, while surplus amounts are stored in the liver. The multi-strain probiotic used in our study contains LAB strains, which colonize the digestive tract of birds and become part of the intestinal microbiome. The accumulation of zinc in the body is significantly influenced by strains of lactic acid bacteria (LAB) [42], which enable the transformation of the inorganic forms of minerals into an organic form in which zinc binds to protein complexes and is absorbed in the small intestine in a manner typical of peptides and proteins [42,43]. This can explain the high zinc concentration in the liver of birds additionally receiving a multi-strain probiotic (group IV). The Zn-enriched biomass of the microbiome, containing LAB, supplies the body with significant amounts of this element, essential for meeting all vital needs [42,43].

It is also worth emphasizing that the addition of phytase to feed may affect Zn metabolism and its concentration in the body. There are a lot of conflicting data in the available literature regarding the addition of phytase to feed. Świątkiewicz et al. [44] showed that the administration of phytase in feed containing a Zn supplement in organic form reduces the relative bioavailability of Zn. On the other hand, Yi et al. [45] and Thiel and Weigand [46] showed that the addition of phytase to poultry feed resulted in greater Zn retention and reduced its excretion into the environment. Moreover, Augspurger et al. [47] showed that Zn supplementation in the diet of chickens reduces the effectiveness of phosphorus-releasing phytase, which indicates a strong inhibitory effect of Zn on phytate hydrolysis by phytase. These conflicting data require further detailed investigation at the cellular level. In our experiment, Zn supplementation was not used in the first 7 days of life, and Zn-Gly chelate administered in ovo had a significant impact on the metabolic processes of the birds and on the accumulation of Zn in the serum. Understanding the reasons for this effect of organic Zn administered in ovo requires further detailed research.

Previous research indicates that the addition of zinc in organic form to feed for layer hens significantly increases its concentration in the egg yolk and its bioavailability, ensures high productivity during egg production, and improves egg quality [48,49]. Our results indicate that in ovo administration of a multi-strain probiotic and Zn-Gly chelate (group IV) increases the concentration of Zn, which accumulates in the yolk sac and, despite its use by the embryo to build tissues, is still detected in large amounts. Zn supplementation has been shown to increase mRNA expression of CuZnSOD in the liver and its activity in broilers [50], laying hens [48], and other animal species, such as pigs [51]. The results of the present study, obtained in SDS-PAGE and western blot, confirmed the presence of Cu/Zn SOD in the liver and in the small and large intestines at 12 h and 7 days after hatching in all experimental groups and the control. Additional analysis of polyacrylamide gels using GelAnalyzer 19.1 [52] software showed the highest expression of Cu/Zn SOD in the small and large intestine at 12 h post-hatch in the groups of birds receiving the multi-strain probiotic and Zn-Gly chelate (group IV) or Zn-Gly chelate (group III). At 7 days of age, however, the highest expression of Cu/Zn SOD was noted in the small intestine in group IV, receiving the multi-strain probiotic and Zn-Gly chelate, and in the large intestine in group III, receiving Zn-Gly chelate. The high concentration of Cu/Zn SOD in the small intestine of chicks in the early post-hatch period is confirmed by Tang et al. [53], who showed that SOD activity is higher in the small intestine than in other tissues.

The experiment did not show differences in the expression of Cu/Zn SOD in the liver, in agreement with the analyses of the zinc concentration in this organ, which at 12 h post-hatch showed no statistical differences. This is most likely explained by the redistribution of Zn to other tissues undergoing rapid growth and development during this period. 

It is interesting that despite the significant amount of Zn in the yolk sac, the western blot did not show the presence of Cu/Zn SOD.

The mass distribution profiles in the tissue homogenates differed most in the case of the small and large intestines at 12 h post-hatch and at 7 days of age for supplementation with a multi-strain probiotic and Zn-Gly chelate (groups III and IV vs. the other groups). The distributions for these groups consisted of a large number of bands corresponding to nearly the entire range of separated masses.

Screening of molecular weight distributions revealed differences depending on the sampling time and feeding group. Based on mass spectra, we determined changes in specific molecular weights, entire ranges, and soft shifts. Additionally, peak positions interpreted using the Uniprot database were used to assign specific proteins to twelve peaks. These proteins differed in their occurrence and amounts among the groups. All tissues were tested in wide ranges from 600–15,000 Da. The most significant molecular weight ranges are shown in Table 3.

Analysis of the mass spectra of proteins in the liver showed expression of MHC class I antigen, with a molecular weight of 7052 Da, in all experimental groups and the control group at 12 h post-hatch and at 7 days of age; it was highest at 7 days in group II, receiving the multi-strain probiotic in ovo. MHC class I molecules are present on most nucleated cells, and their basic function is the presentation of peptides to cytotoxic CD8+ T lymphocytes [54]. According to Miller and Taylor [55], high expression of MHC class I in chickens is correlated with resistance to infectious diseases. The higher expression of this protein at 7 days of age in chicks is linked to the stimulation of the immune system during embryonic development, with the body’s response to environmental antigens colonizing the body after hatching, and also with vaccinations against infectious diseases after hatching.

Analysis of the MALDI—TOF MS spectra of the liver tissue in all experimental groups and the control at 12 h post-hatch showed expression of the protein puromycin-sensitive aminopeptidase isozyme II (PSA-II), with a molecular weight of 2159 Da. PSA-II is a Zn^2+^ metallopeptidase present in the cytoplasm of the cells of various tissues, mainly the brain [56]. Its function involves the digestion of short polyglutamine peptides [57] and proteasome products to amino acids or peptides, which are then presented as antigens to MHC class I molecules [58]. Identification of this protein demonstrates that during the post-hatch period an immune response is activated which influences the resistance of birds in later life. The lack of expression of this protein in the liver at 7 days of age is surprising; it may be linked to the occurrence of this isoenzyme in other tissues, such as the brain, as shown by Hui et al. [59].

At 12 h post-hatch, only the protein transthyretin (TTR), with a molecular weight of 1116 Da, was identified in the liver of birds receiving Zn-Gly chelate (group III) or Zn in combination with a multi-strain probiotic (group IV). Transthyretin, synthesized in the liver and present in the cerebrospinal fluid and plasma, is responsible for transport of thyroxin (T4) and retinol (vitamin A) [60]. Deficiency of this protein has been shown to impair the distribution of these compounds [60]. On the other hand, an unfavorable trait of transthyretin is its tendency to form amyloid fibers, which are deposited in the tissues and can lead to the development of amyloid diseases [61]. However, all biological functions of TTR in the body are not fully understood. The clear signal obtained in the study indicating synthesis of this protein in groups III and IV may be linked to supplementation with the easily absorbed organic form of Zn.

Another protein for which we observed a change in the MALDI—TOF MS analysis was FMRFamide-like neuropeptide (LPLRF-amide), with a molecular weight of 645 Da. This was present in the liver of all experimental groups and in the control, both at 12 h post-hatch and at 7 days of age. An interesting aspect of this part of the study was the shift of the peak identifying this protein at 7 days of age towards higher weights, which may be indicative of post-translational modifications of the protein during the physiological development of chickens. These neuropeptides are found in both the central and peripheral nervous system, functioning as neurotransmitters and neuromodulators [62].

Examination of the tissues of the small intestine by MALDI—TOF MS showed the presence of the characteristic protein serglycin, with a molecular weight of 14,069 Da, at 12 h post-hatch. This protein was identified in the groups of birds receiving the multi-strain probiotic and Zn-Gly chelate (group IV) or Zn-Gly chelate (group III) in ovo as well as in the control group. Serglycin is a haematopoietic proteoglycan with varied functions depending on the cells in which it is synthesized [63]. The high expression of this protein in groups III and IV may be linked to the administration of easily absorbed Zn chelate, which causes activation of lymphocytes involved in the cellular response and proliferative cytokines released in the intestines [11] or may indicate inflammation developing in the intestines due to the irritant effect of excess Zn on rapidly dividing enterocytes. Expression of this protein was not observed in the chickens receiving the multi-strain probiotic (group II), which demonstrates that the strains contained in it stabilize the intestinal environment, maintaining homeostasis. Another protein identified in the small intestine at 12 h post-hatch, but only in the control group (group I), was insulin 2, with a molecular weight of 5065 Da. According to the UniProt database [64], this protein is a fragment of insulin and the product of its breakdown in the small intestine. Its expression in the control group may be due to the cells’ greater need for energy substrates essential to growth and development. The lack of its expression in the experimental groups suggests that in ovo supplementation with preparations regulating glycogenesis at the cell level is effective. At 7 days of age, in the chickens receiving Zn-Gly chelate in ovo and in the control group, we also observed expression of mitochondrial inner membrane protease subunit 2, with a molecular weight of 11,296 Da. This protein is one of the mitochondrial proteases, which regulate numerous functions of the mitochondria, processes taking place in them, and their programmed death.

MALDI—TOF MS analysis of the large intestinal tissues showed expression of glutamate-cysteine ligase (gamma-ECS, gamma-glutamylcysteine synthetase), with a molecular weight of 8171 Da, at 12 h post-hatch in the experimental groups and in the control, with the highest expression shown in the chickens in group II, which received the multi-strain probiotic in ovo. This protein is the first enzyme of the biosynthesis pathway of cellular glutathione (GSH), which takes part in antioxidant processes [65]. Expression of this protein in the study groups confirms the protective role of an in ovo administered multi-strain probiotic and Zn in oxidative stress, which is important for cell survival and embryo development [66].

The protein V-type proton ATPase subunit, with a molecular weight of 9002 Da, was expressed in the tissues of the large intestine of the control group at 12 h post-hatch. We also showed expression of this protein at 12 h post-hatch in the yolk sac in all groups, with the highest expression shown in the yolk sac of the birds receiving the multi-strain probiotic and Zn-Gly chelate in ovo (group IV). This protein performs numerous functions, including in pH regulation, leads to acidification of intracellular compartments, is responsible for proton transport from the cytosol to the extracellular space, and also takes part in neoplastic and neurodegenerative processes [67,68]. An important function of this protein is its involvement in maintaining the homeostasis of nutrients and energy in cells [69].

Differences in the peptide and protein composition of the test samples were least evident in the case of the yolk sacs. Differences were shown in the intensity of peaks with molecular weights of 9152 Da, corresponding to epidermal differentiation protein (NK2 homeobox 3); 9678 Da, corresponding to prolactin-releasing hormone; and 8433 Da, corresponding to protein-serine/threonine phosphatase. The concentrations of these peptides were shown to increase significantly following in ovo supplementation with a multi-strain probiotic and Zn-Gly chelate (group IV) or Zn-Gly chelate (group III). Literature data indicate that these proteins perform numerous functions in the body, including involvement in cell differentiation, the cell cycle, regulation of the immune response, metabolic processes, growth, development, and reproduction [70,71].

## 5. Conclusions

In summary, we have demonstrated that in ovo supplementation of Zn-Gly chelate alone and in combination with a multi-strain probiotic has a significant impact on Zn metabolism in the embryo and newly hatched chicks, which is reflected in increased concentration of Zn in the serum and yolk sac. It is believed that the accumulation of zinc in the yolk sac and gastrointestinal tract is associated with a protective function for the embryo and chicks through the element’s participation in antioxidant defense processes. Both dietary supplements also contributed to an increase in zinc concentration in the liver in birds aged 7 days. Additionally, analysis of the MALDI—TOF MS spectra of the tissues of chickens showed in all experimental groups expression of different proteins and peptides presumably performing numerous and diverse functions in the chicks during the post-hatching period, including regulation of the immune response, metabolic processes, growth, development, and reproduction. It is worth noting that differences in protein expression depended on the type of diet supplement, the type of tissues analyzed, and when the analyses were performed. However, understanding of the mechanisms underlying these differences will require further research with the use of imaging of the proteome of individual tissues and identification of differentiating proteins by MALDI—TOF MS.

## Figures and Tables

**Table 1:** Validation parameters used in the Zn assay.

Element/Method	Zn/FAAS ^1^
Flame/assay technique	acetylene/air/absorption
Wavelength [nm]	213.9
Slit width [nm]	1.0
Lamp current [mA]	5.0
LOD—limit of detection [mg/kg]	0.4
LOQ—limit of quantitation [mg/kg]	0.9
Precision (coefficient of variation) (%)	5.1
Recovery of the CRM (%)	106
Expanded uncertainty [%]	14

^1^ FAAS: flame atomic absorption spectrometry.

**Table 2:** Effect of the main factors and their interactions on zinc level in serum and studied tissues [mg/kg] at 12 h and 7 days after hatching.

Effect	Serum		Liver		Small Intestine		Yolk Sac
	12 h	7 Days	12 h	7 Days	12 h	7 Days	12 h
Prob+ (group II)	1.57 ± 0.20	2.99 ± 0.31	18.40 ± 2.35	22.8 ± 0.53	23.63 ± 2.83	27.35 ± 0.33	14.98 ± 1.59
	F = 2.75	F = 25.74	F = 9.42	F = 0.0	F = 0.10	F = 8.40	F = 0.50
	*p* = 0.11	*p* < 0.001	*p* < 0.001	*p* = 0.77	*p* = 0.76	*p* < 0.001	*p* = 0.49
	η^2^ = 0.12%	η^2^ = 56.27%	η^2^ =29.4%	η^2^ = 0.44%	η^2^ =0.47%	η^2^ = 8.32%	η^2^ = 0.31%
Zn-Gly+ (group III)	2.20 ± 0.30	2.93 ± 0.22	21.00 ± 3.43	34.73 ± 7.14	24.67 ± 2.33	24.85 ± 1.56	32.82 ± 4.76
	F = 1375.24	F = 0.25	F = 1.78	F = 131	F = 0.29	F = 58.64	F = 94.43
	*p* < 0.001	*p* = 0.63	*p* = 0.2	*p* < 0.001	*p* = 0.59	*p* < 0.001	*p* < 0.001
	η^2^ = 60.11%	η^2^ = 1.22%	η^2^ = 5.50%	η^2^ = 86.77%	η^2^ = 1.39%	η^2^ = 61.62%	η^2^ = 60.34%
Zn-Gly+Prob+ (group IV)	13.88 ± 1.69	2.58 ± 0.26	23.30 ± 2.28	30.85 ± 0.56	24.43 ± 2.16	24.78 ± 1.09	25.12 ± 2.82
	F = 889.93	F = 1.23	F = 1.03	F = 3.0	F = 0.28	F = 8.13	F = 41.56
	*p* < 0.001	*p* = 0.29	*p* = 0.32	*p* = 0.12	*p* = 0.60	*p* < 0.001	*p* < 0.001
	η^2^ = 38.90%	η^2^ = 5.78%	η^2^ =3.2%	η^2^ = 11.77%	η^2^ = 96.77%	η^2^ = 8.54%	η^2^ = 12.78%

Results of the two-way analysis of variance (ANOVA). Zn-Gly+, zinc-glycine chelate; Prob+, multi-strain probiotic; Zn-Gly+Prob+, interaction zinc-glycine chelate and multi-strain probiotic; η^2^, correlation ratio; F, ratio of variance; *p*, significance level.

**Table 3:** Protein identification by UniProt Entry according to their molecular weight in chicken tissues.

Protein Name	Weight (Da)	UniProt Entry
Liver		
FMRFamide-like neuropeptide	645	P83308
Transthyretin	1117	Q6LAP1
Puromycin-sensitive aminopeptidase isozyme II	2162	Q9PS17
MHC class I antigen	(7052.50)/7053	Q4ZGL8
Small intestine		
Insulin 2	5067	A0A3G3C4S4
Mitochondrial inner membrane protease subunit 2	11,298	A0A8V0X568
Serglycin	14,070	A0A8V0ZRY6
Large intestine		
Glutamate—cysteine ligase	8172	A0A8V0X4D6
V-type proton ATPase subunit	9008	Q5ZJC4
Yolk sac		
Protein-serine/threonine phosphatase	8433	A0A3Q2U207
V-type proton ATPase subunit	9002	Q5ZJC4
Epidermal differentiation protein	9152	A0A088BHA8
Prolactin-releasing hormone	9678	A0A1D5PYU4

**Table 4:** Mean SOD level determined by western blot—band volume according to GelAnalyzer 19.1.

Time	Tissue	SOD Band Volume	SD (±)	Time	Tissue	SOD Band Volume	SD (±)
Liver				Liver			
12 h	I	901	4.32	7 days	I	859	3.83
	II	940	4.37		II	751	1.87
	III	994	3.85		III	686	4.08
	IV	1034	6.06		IV	765	4.08
Small intestine				Small intestine			
12 h	I	977	8.89	7 days	I	926	2.34
	II	844	4.04		II	445	6.28
	III	1574	3.83		III	683	5.89
	IV	1788	2.16		IV	1537	5.01
Large intestine				Large intestine			
12 h	I	406	5.09	7 days	I	1672	6.92
	II	297	2.88		II	421	8.01
	III	817	3.50		III	1819	6.10
	IV	911	3.08		IV	976	5.16

SD—standard deviation.

## Data Availability

All data generated or analyzed during this study are included in this published article, and are available on request from the corresponding author.

## Supplementary Materials

The following supporting information can be downloaded at: https://www.mdpi.com/article/10.3390/cimb46020080/s1, Table S1: Results of two-way ANOVA for the effect of zinc-glycine chelate (group III, Zn-Gly+) and multi-strain probiotic (group II, Prob+) and interaction of Zn-Gly+Prob+ (group IV) supplementation on zinc level in selected tissues at 12 h and 7 days after hatching. Figure S1: SDS-PAGE protein gels stained with Coomassie brilliant blue. The gels contain proteins from chicken large intestine. I—control group (Zn-Gly-Prob-; eggs injected with 0.9% physiological saline solution); group II (Prob+)—eggs injected with a multi-strain probiotic; group III (Zn-Gly+)—eggs injected with zinc glycine chelate; group IV (Zn-Gly+Prob+)—eggs injected with a multi-strain probiotic and zinc glycine chelate. Figure S2: SDS-PAGE protein gels stained with silver. The gels contain proteins from chicken large intestine. I—control group (Zn-Gly-Prob-; eggs injected with 0.9% physiological saline solution); group II (Prob+)—eggs injected with a multi-strain probiotic; group III (Zn-Gly+)—eggs injected with zinc glycine chelate; group IV (Zn-Gly+Prob+)—eggs injected with a multi-strain probiotic and zinc glycine chelate. Figure S3: SDS-PAGE protein gels stained with Coomassie brilliant blue. The gels contain proteins from chicken small intestine. I—control group (Zn-Gly-Prob-; eggs injected with 0.9% physiological saline solution); group II (Prob+)—eggs injected with a multi-strain probiotic; group III (Zn-Gly+)—eggs injected with zinc glycine chelate; group IV (Zn-Gly+Prob+)—eggs injected with a multi-strain probiotic and zinc glycine chelate. Figure S4: SDS-PAGE protein gels stained with silver. The gels contain proteins from chicken small intestine. I—control group (Zn-Gly-Prob-; eggs injected with 0.9% physiological saline solution); group II (Prob+)—eggs injected with a multi-strain probiotic; group III (Zn-Gly+)—eggs injected with zinc glycine chelate; group IV (Zn-Gly+Prob+)— eggs injected with a multi-strain probiotic and zinc glycine chelate. Figure S5: SDS-PAGE protein gels stained with Coomassie brilliant blue. The gels contain proteins from chicken yolk sac. I—control group (Zn-Gly-Prob-; eggs injected with 0.9% physiological saline solution); group II (Prob+)—eggs injected with a multi-strain probiotic; group III (Zn-Gly+)—eggs injected with zinc glycine chelate; group IV (Zn-Gly+Prob+)— eggs injected with a multi-strain probiotic and zinc glycine chelate. Figure S6: SDS-PAGE protein gels stained with silver. The gels contain proteins from chicken yolk sac. I—control group (Zn-Gly-Prob-; eggs injected with 0.9% physiological saline solution); group II (Prob+)—eggs injected with a multi-strain probiotic; group III (Zn-Gly+)—eggs injected with zinc glycine chelate; group IV (Zn-Gly+Prob+)—eggs injected with a multi-strain probiotic and zinc glycine chelate. Figure S7: Western blot with SOD band extracted from chicken large intestine. I—control group (Zn-Gly-Prob-; eggs injected with 0.9% physiological saline solution); group II (Prob+)—eggs injected with a multi-strain probiotic; group III (Zn-Gly+)—eggs injected with zinc glycine chelate; group IV (Zn-Gly+Prob+)—eggs injected with a multi-strain probiotic and zinc glycine chelate. Figure S8: Western blot with SOD band extracted from chicken liver. I—control group (Zn-Gly-Prob-; eggs injected with 0.9% physiological saline solution); group II (Prob+)—eggs injected with a multi-strain probiotic; group III (Zn-Gly+)—eggs injected with zinc glycine chelate; group IV (Zn-Gly+Prob+)—eggs injected with a multi-strain probiotic and zinc glycine chelate. Figure S9: Western blot with SOD band extracted from chicken yolk sac. I—control group (Zn-Gly-Prob-; eggs injected with 0.9% physiological saline solution); group II (Prob+)—eggs injected with a multi-strain probiotic; group III (Zn-Gly+)—eggs injected with zinc glycine chelate; group IV (Zn-Gly+Prob+)—eggs injected with a multi-strain probiotic and zinc glycine chelate.
