# Supplementary material for: Proteome and Peptidome Changes and Zn Concentration in Chicken after In Ovo Stimulation with a Multi-Strain Probiotic and Zn-Gly Chelate: Preliminary Research
Source: Curr Issues Mol Biol. 2024 Feb 1;46(2):1259–80. doi: 10.3390/cimb46020080 (PMC10888147; doi:10.3390/cimb46020080)
Supplement: Supplementary file 1 [file cimb-46-00080-s001.zip › Suplementary Table S1.pdf]

current issues in molecular biology

MDPI

Table S1. Results of two-way ANOVA for the effect of zinc-glycine chelate (group III, Zn-Gly+) and multi-strain probiotic (group II, Prob+) and interaction Zn-Gly+Prob+ (group IV) supplementation on zinc level in selected tissues at 12 hours and 7 days after hatching.

Effect/Interaction	**Serum**									
	**12 h**					**7 days**				
	**SS**	**df**	**MS**	**F**	***p***	**SS**	**df**	**MS**	**F**	***p***
Corrected model	8.68	1	8.68	894.59	>0.01	0.56	1	0.56	92.59	>0.01
Zn-Gly+ (group III)	13.35	1	13.35	1375.24	>0.01	0.00	1	0.00	0.25	0.62
Prob+ (group II)	0.03	1	0.03	2.75	0.11	0.16	1	0.16	25.74	>0.05
Zn-Gly+Prob+ (group IV)	8.64	1	8.64	889.93	>0.01	0.01	1	0.01	1.23	0.28
Error	0.19	20	0.01	0.00		0.12	20	0.01	0.00	
	**Liver**									
Corrected model	3886.47	1	3886.47	1969.23	>0.01	210.56	1	210.56	901423484.00	>0.01
Zn-Gly+ (group III)	3.51	1	3.51	1.78	0.19	0.00	1	0.00	131.00	>0.01
Prob+ (group II)	18.73	1	18.73	9.49	>0.05	0.00	1	0.00	0.00	0.77
Zn-Gly+Prob+ (group IV)	2.04	1	2.04	1.03	0.32	0.00	1	0.00	3.00	0.12
Error	39.47	20	1.97	0.00		0.00	20	0.00	0.00	
	**Small intestine**									
Corrected model	543.17	1	543.17	11675.75	>0.01	2.487035E+41	1	2.487035E+41	126.48	>0.01
Zn-Gly+ (group III)	0.01	1	0.01	0.29	0.60	1.152930E+41	1	1.152930E+41	58.63	>0.01
Prob+ (group II)	0.00	1	0.00	0.10	0.76	1.651029E+40	1	1.651029E+40	8.40	>0.05
Zn-Gly+Prob+ (group IV)	0.01	1	0.01	0.28	0.60	1.597927E+40	1	1.597927E+40	8.13	>0.05
Error	0.93	20	0.05	0.00		3.932776E+40	20	1.966388E+39		
	**Yolk sac**									
Corrected model	426.10	1	426.10	9803.90	>0.01					
Zn-Gly+ (group III)	4.10	1	4.10	94.43	>0.01					
Prob+ (group II)	0.02	1	0.02	0.50	0.5					
Zn-Gly+Prob+	1.81	1	1.81	41.56	>0.01					

(group IV)			
Error	0.87	20	0.04

SS – sum of squares; df – degrees of freedom; MS – mean square; F – ratio of variance, *p* – significance level.
