# Supplementary figures and images for: Proteome and Peptidome Changes and Zn Concentration in Chicken after In Ovo Stimulation with a Multi-Strain Probiotic and Zn-Gly Chelate: Preliminary Research
Source: Curr Issues Mol Biol. 2024 Feb 1;46(2):1259–80. doi: 10.3390/cimb46020080 (PMC10888147; doi:10.3390/cimb46020080)

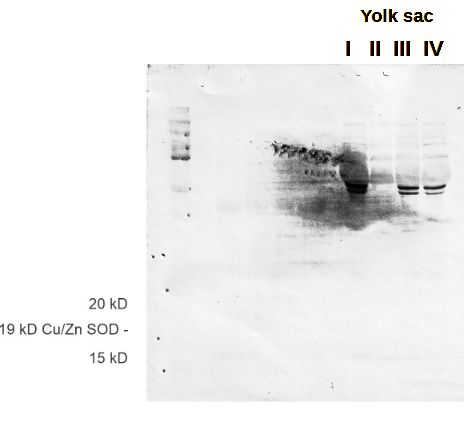

Supplement: Supplementary file 1 [file cimb-46-00080-s001.zip › Fig S9 WB yolk sac.jpg]

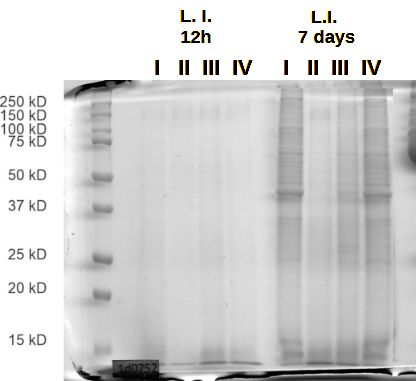

Supplement: Supplementary file 1 [file cimb-46-00080-s001.zip › Fig. S1 L.I_coomassie.jpg]

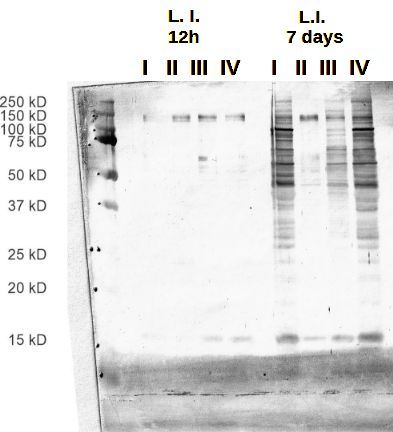

Supplement: Supplementary file 1 [file cimb-46-00080-s001.zip › Fig. S2 L.I_silver.jpg]

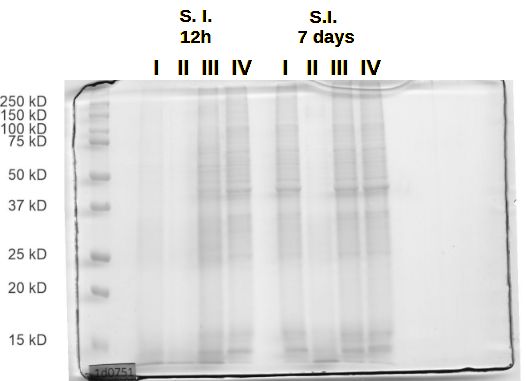

Supplement: Supplementary file 1 [file cimb-46-00080-s001.zip › Fig. S3 S.I_coomassie.jpg]

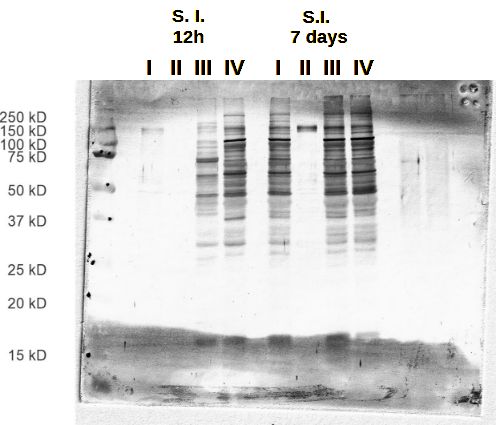

Supplement: Supplementary file 1 [file cimb-46-00080-s001.zip › Fig. S4 S.I_silver.jpg]

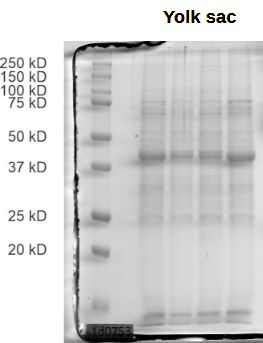

Supplement: Supplementary file 1 [file cimb-46-00080-s001.zip › Fig. S5 Y.S_coomassie.jpg]

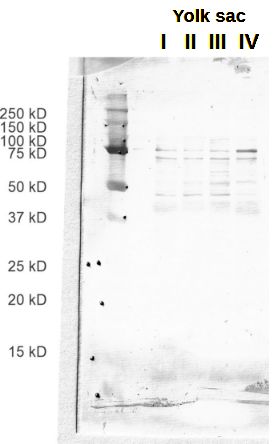

Supplement: Supplementary file 1 [file cimb-46-00080-s001.zip › Fig. S6 Y.S_silver.jpg]

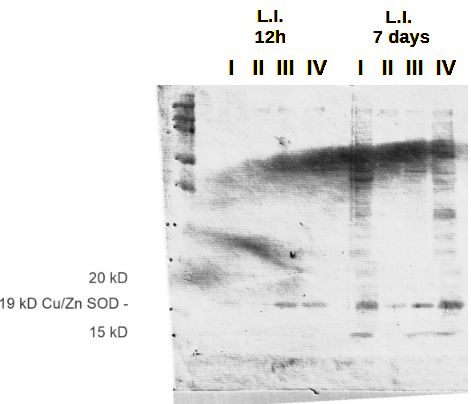

Supplement: Supplementary file 1 [file cimb-46-00080-s001.zip › Fig. S7 WB L.I.jpg]

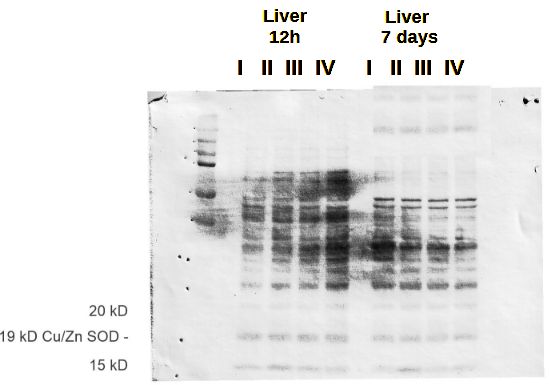

Supplement: Supplementary file 1 [file cimb-46-00080-s001.zip › Fig. S8 WB Liver.jpg]
